# Advancing passive BCIs: a feasibility study of two temporal derivative features and effect size-based feature selection in continuous online EEG-based machine error detection

**DOI:** 10.3389/fnrgo.2024.1346791

**Published:** 2024-05-15

**Authors:** Yanzhao Pan, Thorsten O. Zander, Marius Klug

**Affiliations:** ^1^Chair of Neuroadaptive Human-Computer Interaction, Brandenburg University of Technology Cottbus-Senftenberg, Cottbus, Germany; ^2^Young Investigator Group – Intuitive XR, Brandenburg University of Technology Cottbus-Senftenberg, Cottbus, Germany

**Keywords:** Brain Computer Interface, human-robot interaction, error detection, signal processing, feature extraction, time-derivative features, artifact rejection

## Abstract

The emerging integration of Brain-Computer Interfaces (BCIs) in human-robot collaboration holds promise for dynamic adaptive interaction. The use of electroencephalogram (EEG)-measured error-related potentials (ErrPs) for online error detection in assistive devices offers a practical method for improving the reliability of such devices. However, continuous online error detection faces challenges such as developing efficient and lightweight classification techniques for quick predictions, reducing false alarms from artifacts, and dealing with the non-stationarity of EEG signals. Further research is essential to address the complexities of continuous classification in online sessions. With this study, we demonstrated a comprehensive approach for continuous online EEG-based machine error detection, which emerged as the winner of a competition at the 32nd International Joint Conference on Artificial Intelligence. The competition consisted of two stages: an offline stage for model development using pre-recorded, labeled EEG data, and an online stage 3 months after the offline stage, where these models were tested live on continuously streamed EEG data to detect errors in orthosis movements in real time. Our approach incorporates two temporal-derivative features with an effect size-based feature selection technique for model training, together with a lightweight noise filtering method for online sessions without recalibration of the model. The model trained in the offline stage not only resulted in a high average cross-validation accuracy of 89.9% across all participants, but also demonstrated remarkable performance during the online session 3 months after the initial data collection without further calibration, maintaining a low overall false alarm rate of 1.7% and swift response capabilities. Our research makes two significant contributions to the field. Firstly, it demonstrates the feasibility of integrating two temporal derivative features with an effect size-based feature selection strategy, particularly in online EEG-based BCIs. Secondly, our work introduces an innovative approach designed for continuous online error prediction, which includes a straightforward noise rejection technique to reduce false alarms. This study serves as a feasibility investigation into a methodology for seamless error detection that promises to transform practical applications in the domain of neuroadaptive technology and human-robot interaction.

## 1 Introduction

In recent years, the field of human-robot collaboration has brought in the necessity for assistive robots to harmoniously integrate into human environments, a goal achievable through human-intention estimation based on sensor data (Dani et al., 2020). A frontier that is emerging as particularly promising is the incorporation of Brain Computer Interfaces (BCI), which can provide feedback on the user state to adapt interactions dynamically. In doing so, they pave the way for a more synergistic and intuitive human-robot collaboration, e.g., by enhancing the functionalities of exoskeletons and orthoses (Kirchner and Bütefür, 2022). Among the notable applications in this domain, the use of error-related potentials (ErrPs) as implicit feedback has been successfully validated in real robot scenarios (Kim et al., 2017). By continuously extracting information from spontaneous brain activity and using it as implicit feedback to assist ongoing tasks, this passive BCI approach can facilitate more user-centered human-machine interactions (Zander and Kothe, 2011).

A series of studies have focused on the continuous online detection of ErrPs through various approaches. Spüler and Niethammer (2015) illustrated that ErrPs can be measured and further classified in a continuous manner during a cursor control task. Omedes et al. (2014) introduced a method that continuously detected error potentials during device operation. Kim et al. (2023) demonstrated that asynchronous detection of ErrP over several seconds is possible in human-robot interactions, despite the challenge of high false alarms in longer task durations. Lopes-Dias et al. (2021) validated the feasibility of detecting ErrPs in an online experiment without offline calibration. It's important to note that each of these studies used their unique criteria and different datasets for evaluating online performances, thereby rendering a direct comparison of their classification results unfeasible. However, a common thread in their endeavors was the pursuit of a delicate equilibrium between false alarms and misses, highlighting the complexity of continuous classifications in online sessions (Liu et al., 2018).

Nowadays, various modern classification algorithms have been developed in Electroencephalography (EEG)-based BCIs, along with novel feature types to represent EEG signals in a compact way (Lotte et al., 2018), such as connectivity features (Wei et al., 2007), complexity measures (Brodu et al., 2012), and tensors (Congedo et al., 2017). Some studies have shown that the combination of different feature types generally contributes to higher classification accuracies than using a single feature type (Dornhege et al., 2004; Brodu et al., 2012; Roy et al., 2016). Therefore, more feasible feature types should be explored for EEG-based BCIs, particularly those with low computational demands suitable for online applications. Moreover, since some features extracted from EEG signals may be redundant, it is crucial to explore associated feature selection techniques that can reduce dimensionality and thus produce faster online predictions, as well as simplify the observation of which features are actually related to the targeted mental states (Lotte et al., 2018).

Previously, Andreou and Poli (2016) explored the potential of using first-order temporal derivatives of EEG signals as inputs to BCIs, an approach rarely used in the field. Their research using a P300-based mouse BCI setup showed that the use of these features improved classification accuracy, both in combination with EEG amplitudes and even more so when used independently. More research is needed in this feature type. Furthermore, to our knowledge, few studies have investigated second-order time-derivative features, which we hypothesize could be effective in capturing subtle fluctuations or trends that might not be visible through the first-order derivatives. There is a lack of feasibility studies exploring the individual and combined use of these two time-derivative features, particularly in the comprehensive capture of the complex patterns present in EEG data.

Additionally, in real-world scenarios of continuous online EEG-based detection of machine errors, ongoing EEG signals are continuously acquired, processed, and then classified with a pre-trained model as quickly and accurately as possible. Beyond an effective pattern recognition approach, two major challenges should be considered. One is to address the non-stationarity in EEG signals, which refers to changes over time and can have a major impact on BCI classification with prolonged use (Krumpe et al., 2017). Shenoy et al. (2006) quantified this change and showed that motor imagery signals used for control can change substantially from offline calibration sessions to online control sessions, and also within a single session. Christensen et al. (2012) applied three popular pattern classification techniques to EEG data from participants executing a complex multi-task over a span of 5 days in a month, observing a significant decrease in classification accuracy across different days. Although adaptive strategies (Shenoy et al., 2006) and a few calibration-free online classification architectures (Grizou et al., 2014; Wimpff et al., 2023) have been proposed for offline to online session transfer, it is crucial to investigate more pattern recognition approaches in a calibration-free manner over longer periods, such as days or weeks. The second challenge is the presence of artifacts in EEG signals during the continuous online classification. Effective artifact removal techniques are crucial to study brain dynamics in a natural everyday environment or during high-intensity motor activities (Gorjan et al., 2022). In the field of Mobile Brain/Body Imaging (Makeig et al., 2009; Gramann et al., 2011; Jungnickel et al., 2019), offline artifact correction methods such as the use of Independent Component Analysis (ICA) have been consistently found to be the most effective tools. But while ICA can be used in mobile settings and can be effective also with lower-density electrode layouts (Klug and Gramann, 2021), it is not directly online-capable. Although online-capable artifact rejection algorithms such as Artifact Subspace Reconstruction (Kothe and Jung, 2015), Online Recursive ICA (Hsu et al., 2014), and Recursive Sparse Bayesian Learning (Ojeda et al., 2018, 2019) have shown potential in specific experimental setups, their performance in continuous online classification—where fast computation is essential—has not been thoroughly investigated. Therefore, there is a clear need for further research into lightweight noise filtering techniques specifically designed for continuous online classification scenarios.

In this study, we present a comprehensive approach for continuous online EEG-based machine error detection that incorporates two temporal-derivative features with an effect size-based feature selection technique for model training, together with a lightweight noise filtering method for non-calibration online sessions on the same test participant. This approach emerged as the overall winner in the competition of “Intrinsic Error Evaluation during Human-Robot Interaction” (IntEr-HRI Competition), a challenge of the 32nd International Joint Conference on Artificial Intelligence (IJCAI 2023). The contribution of our research is 2-fold: first, it validates the feasibility of using two temporal derivative features combined with an associated feature selection approach; second, it presents a framework that seamlessly transitions from offline training on single-trial data to the continuous online classification session 3 months after the initial data collection without further calibration on the same test participant. In particular, in order to reduce false alarms in the process of continuous error prediction, a straightforward yet effective noise rejection technique is proposed.

The following sections are organized as follows: Section 2 provides an overview of the competition, in particular the specific tasks of the challenge, which are further subdivided into the offline and online stages, each with its own problem statement and evaluation metrics. We then present our approach used during the competition and the extended analysis after the competition. In Section 3, we present our findings, again divided into the offline stage, the online stage and the extended analysis. Section 4 analyzes the classification results and provides a discussion on the feasibility of using two temporal derivative features combined with an effect size-based feature selection approach in online EEG-based classification, as well as offline to online session transfer. Finally, the limitations of the study are acknowledged and directions for future research are suggested.

## 2 Competition and methods

### 2.1 Competition management

The competition was organized as the challenge of the 32nd International Joint Conference on Artificial Intelligence^1^ (IJCAI 2023), held in Macau, S.A.R., from August 19–25, 2023. Hosted by the Robotics Innovation Center of the German Research Center for Artificial Intelligence (DFKI) and Universität Duisburg-Essen (UDE), this competition sought to foster innovations in signal processing and machine learning for human-robot interaction, focusing on the continuous detection of erroneous machine behaviors through detailed analysis of human EEG.

The competition was divided into two primary stages: the offline and online stages. Initially, in the offline stage, participating teams utilized a publicly available dataset^2^ recorded from April 24 to 27, 2023, consisting of EEG and Electromyogram (EMG) data from eight individuals assisted in right arm movements by an active orthosis (Kueper et al., 2024). While EMG data was also recorded, this competition only focused on EEG data. Here, teams were tasked with developing machine learning models to identify induced errors using the available labeled EEG samples. The subsequent online stage, held on August 9, 2023, called for the application of these pre-trained models to identify error onsets in real-time, using continuously streamed and unlabeled EEG data during a live session. This approach tested the pre-trained models' efficacy in detecting errors in the orthosis-directed movements as a direct application of the approach developed in the preceding offline stage.

### 2.2 Experimental design

#### 2.2.1 Participants

Eight healthy right-handed volunteers (four males, four females; mean age of 21.8 years) participated in the study. Prior to the experiment, they visited the lab for a brief introduction and preliminary tests including orthosis fitting and EEG cap sizing based on head circumference. Participants, informed of their rights including voluntary withdrawal, gave their written informed consent. The experiment averaged 4.9 h (SD = 0.6 h), with participants compensated at a rate of 10€ per hour.

#### 2.2.2 Experimental setup and procedure

**Figure 1:**
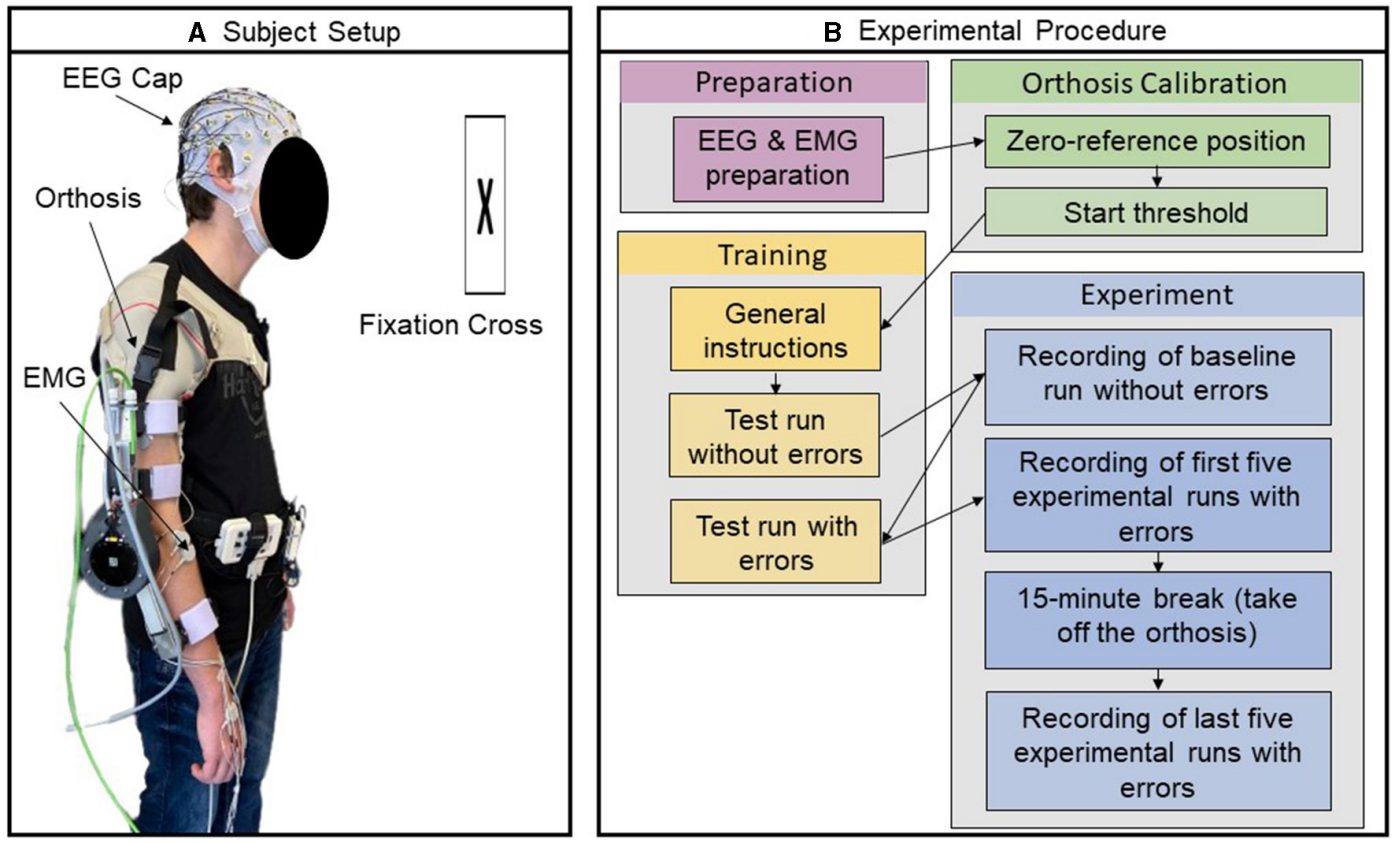
Experimental setup and procedure. **(A)** Participant prepared with EEG and EMG electrodes wearing orthosis on their right arm **(B)** Visualization of the different steps in the experimental procedure. Reprinted from Kueper et al. (2024).

As detailed in Section 2.1, the experimental session and data recording were conducted by the competition organizers (Kueper et al., 2024). Participants were equipped with a 64-channel EEG system and an eight-channel EMG system. They were also fitted with an active orthosis on their right arm, as shown in Figure 1A, and held an air-filled ball in their left hand. The functioning of the orthosis was initiated by the participants applying a force exceeding a specified start threshold in the intended movement direction.

The core task for the participants was to identify errors introduced in the functioning of the orthosis during a series of movement trials comprising both flexion and extension movements. An error referred to a temporary directional change in the orthosis' movement for a short duration (250 ms). During the trial, if the orthosis was amid executing a flexion movement, the introduction of an error would momentarily shift its action to perform an extension, before reverting to continue the flexion trajectory; conversely, it would temporarily switch to a flexion if it was initially undertaking an extension. The first run of the experiment, intended to establish a baseline, involved 30 error-free movement trials. Following this, the participants underwent a training session to familiarize themselves with the sensation of the errors and the corresponding response—squeezing the ball in their left hand. In subsequent experimental runs, six errors were randomly introduced in a series of 30 movement trials. The experiment involved 10 such runs with randomized error sequences, and the participants were asked to maintain specific postures and gaze directions to minimize data artifacts. Accordingly, the experimental design anticipated a cumulative total of 480 responses to error events, the product of six error events per dataset, 10 datasets, and eight participants. An overview of the procedure is visualized in Figure 1B. After the experiment, competition organizers assessed the response time (RT) by analyzing the duration from the occurrence of the error event to the participants' response. The median RT was calculated for each individual over 10 datasets. Subsequently, the mean (μ) and standard deviation (σ) of these median RTs were calculated across all participants, resulting in a value of 0.75 ± 0.11 (μ ± σ) seconds.

#### 2.2.3 Data acquisition

The data acquisition process encompassed the recording of EEG data and the recording of EMG data. The EEG data was acquired using the 64-channel LiveAmp system coupled with an ActiCap slim electrode system functioning with an extended 10–20 layout, both provided by Brain Products GmbH. The reference electrode was positioned at FCz and the ground at AFz. To ensure the high quality of the EEG data, the impedance of all 64 electrodes was kept below a threshold of 5 kΩ, a criterion upheld through checks conducted before and after each experimental run. The EEG data was sampled at a frequency of 500 Hz using Brain Products GmbH's Recorder software (version 1.25.0001), which employed hardware filters to restrict the data bandwidth to a passband range of 0.0–131.0 Hz.

While EMG recording was part of the data acquisition process, the focus of this competition and the subsequent analysis was the EEG data. Therefore, details pertaining to EMG recording will not be covered in this paper. For an in-depth exploration of EMG recording and its synchronization with EEG data, see Kueper et al. (2024).

The acquired EEG data is structured according to the BrainVision Core Data Format 1.0. This format involves three key files: a binary data file (.eeg), a header file (.vhdr), and a marker file (.vmrk). The marker files (.vmrk), stored in each participant's EEG data folder, document all significant events occurring during each set. Specific markers denote the commencement of flexion and extension movements, represented by S64 and S32, respectively. Error-free trials are indicated by S48 markers, positioned around the calculated mean error site, representing the average angle at the onset of error stimuli during the flexion or extension trials that incorporate errors. The S96 event marker is triggered as soon as an error is introduced during the trial. Moreover, the act of the participant squeezing the ball initiates the recording of the S80 event in the marker file.

In the online stage, the experimental procedure mirrored the process utilized in the offline stage, but with the critical addition of real-time data streaming. To this end, the setup involved a secure connection to a private network via a VPN tunnel facilitated through the Wireguard VPN Tunnel tool. Additionally, the Lab Streaming Layer (LSL) was employed to manage a 65-channel data stream, which included 64 EEG data channels and a specific marker channel, to secure synchronous data transmission. Participating teams were expected to report detected errors swiftly through IP requests. This coordinated approach safeguarded the data and assured uninterrupted real-time data streaming.

### 2.3 Challenge tasks

#### 2.3.1 Offline stage

##### 2.3.1.1 Problem statement

In the initial phase of the offline stage, teams were given pre-recorded single-trial EEG datasets segmented into labeled training data derived from eight participants, each offering eight distinct labeled training sets. Additionally, two unlabeled sets were provided for preliminary model evaluations. Teams were tasked to train machine learning models capable of detecting the onset of the deliberately introduced errors within the data. Validating the model's performance required a 10-fold cross-validation to be executed individually on each participant's training dataset. Subsequently, the teams would test the trained model using the two unlabeled test datasets as a preparation for the real-time scenarios in the upcoming online stage where event markers would be absent.

##### 2.3.1.2 Evaluation metrics

The evaluation metrics for the offline stage focused exclusively on the testing phase, wherein teams were required to simulate an online prediction using the two unlabeled sets. Participating teams were required to submit exactly six sample indices of predicted error onsets per set, which were then converted to respective time points in milliseconds. These time points were then compared against the ground truth reference to calculate the temporal deviation in milliseconds, following a defined limit set within a 1,000-millisecond window after the true stimulus onsets. Any detection occurring outside the 1,000 milliseconds span was not acknowledged as a true positive. Such occurrences were recorded as 1,000 milliseconds deviations, contributing to the team's aggregate score. This score, a summation of temporal deviations accumulated across all sets and participants, yielded a grand total which was used to identify the winner in this stage.

#### 2.3.2 Online stage

##### 2.3.2.1 Problem statement

In the online stage of the competition, selected teams from the offline stage used their models remotely in real time using continuously streamed unlabelled EEG data from one participant who was also part of the offline stage. The same experimental setup as in the offline stage was used. However, the protocol involved two differences: (1) In each set, there were five errors introduced among 30 trials, as opposed to the six errors out of 30 in the offline stage. (2) Two different scenarios were used, each consisting of two sets, resulting in a total of four sets for the online stage. In the first scenario, error detection prompted a direct physical response, which involved pressing an air-filled ball, equivalent to the scenarios of the offline stage. Conversely, the second scenario required awareness of the errors without a physical response being necessitated. The results of the online prediction in both scenarios were evaluated to determine the final winner of the competition.

##### 2.3.2.2 Evaluation metrics

There were two different metrics that were used to determine the final performance score (FPS) for each team: Balanced Accuracy (BA) and consolidated time score (t_score_), which was derived from two additional quantities—the difference between the predicted error time and the ground truth (t_err_), and the computation time (t_comp_). The final performance score was calculated as a weighted average of the BA and t_score_, with a 70% weightage given to the BA. In the online stage, the team with the highest FPS was determined as the winner. The detailed calculation rules for these metrics are provided below.

The BA was derived from the four terms of confusion matrix: True Positive (TP), True Negative (TN), False Positive (FP), and False Negative (FN). The BA was formulated as the mean of the True Positive Rate (TPR, also known as sensitivity) and the True Negative Rate (TNR, also known as specificity), calculated as:


$$\begin{array}{l} BA=\frac{\frac{n_{TP}}{n_{TP}+\;n_{FN}}+\;\frac{n_{TN}}{n_{TN}+\;n_{FP}}}{2} \end{array}$$

Note that the calculation of BA took place within the specified boundaries of individual movement trials, excluding predictions beyond these intervals. Furthermore, in the trials where an error was introduced, the defined limit set within a 1,000-millisecond window after the true error onset was used to evaluate TP and FN. Conversely, any temporal frame situated outside of this specified window within the same trial was used to evaluate TN and FP. This essentially indicates that a trial containing an error that was correctly identified within the specified 1,000-millisecond window, and carried no other predictions outside this window, was recognized both as a TP and a TN. To clarify, this evaluation approach, distinguishing TP and TN within the same trial, was specific to this competition and differed from standard binary classifications. Developed by the competition committee for one-class classification (“error” vs. “the rest”), this approach adopted more stringent criteria to ensure that the system can accurately detect errors within a specified timeframe while minimizing false alarms outside this period. Additionally, if more than one error was predicted in rapid succession, the one closest after the true error was selected by the committee.

The *t*_score_ was calculated based on t_err_ and *t*_comp_. The *t*_err_ represented the deviation in milliseconds between the predicted and actual error onset times, consistent with the metric used in the offline stage. Thus, *t*_err_ was in the range of 0–1,000 ms, as higher values would count as misses. On the other hand, *t*_comp_ indicated the duration required for the classifier model to identify an error once the sample was acquired, underlining the importance of low error detection time. Predictions exceeding a 3,000-millisecond threshold wouldn't qualify as a TP. The initial step in deriving the *t*_score_ involved determining individual time scores (*t*_*i*_) for each TP through logistic regression using t_comp_ as an input, where *f* (0) = *t*_err_ and *f* (3,000) = 1,000. This was followed by aggregating and normalizing these scores to obtain the t_score_, as detailed in the subsequent formula:


$$t_{score}=1-\;\frac{\sum_{i=1}^{n_{TP}}t_{i}}{n_{TP}\cdot 1000}$$

This means that a *t*_score_ of 1 indicates a scenario where the predicted and actual error onset times are identical, coupled with no computational delay. Conversely, a *t*_score_ of 0 indicates a situation where, for each predicted error, either the computation time reaches 3,000 ms or the discrepancy between the predicted and actual error onset times reaches 1,000 ms. Scores in between 0 and 1 have no clear interpretation, but higher scores indicate a quicker detection.

### 2.4 Hardware and software specifications

In both the offline classification and online prediction stages, the software components were executed on a system equipped with the following hardware specifications: an AMD Ryzen 5 3600X 6-Core Processor operating at a clock frequency of 3.80 GHz, 16 GB of RAM. In the offline stage, MATLAB R2021a and the EEGLAB 2022.1 toolbox were used for classifier training, online prediction simulation, in-depth exploration of EEG data, and figure creation (Delorme et al., 2011). Before the online stage, the same classifier was trained using Python 3.8.8, employing a method consistent with the one implemented in MATLAB, with a slight variation in filter selection. The following modules were used to streamline the training process: Scikit-learn (v1.3.0), MNE-Python (v1.4.2) for EEG data reading and preprocessing, imbalanced-learn (v0.11.0), which managed imbalanced datasets adeptly, and NumPy (v1.24.4). In the online stage, the Signal processing module (v1.10.1) was applied for data filtering. The PyLSL module (v1.16.2) enabled the use of Lab Streaming Layer (LSL), which was the data streaming protocol used in the challenge and is generally widely used in electrophysiological and specifically BCI research (Wang et al., 2023).

### 2.5 Methods

#### 2.5.1 Offline stage

As outlined in Section 2.3.1.1, this stage was structured into two primary phases: classifier training using labeled data, and the simulation of online predictions on unlabeled data to test the trained classifier.

##### 2.5.1.1. Classifier training

In the initial step of the classifier training phase, the EEG data was preprocessed by re-referencing to an average reference and applying a zero-phase, non-causal Hamming windowed-sinc FIR highpass [0.1 Hz passband edge, 0.1 Hz transition bandwidth, 0.05 Hz cutoff frequency (−6 db)] and lowpass filter [15 Hz passband edge, 3.75 Hz transition bandwidth, 16.875 Hz cutoff frequency (−6 db)] in succession, using the EEGLAB pop_eegfiltnew function. Subsequently, the data was segmented into epochs of interest, spanning from 100 ms before to 1,000 ms after the stimulus onset. For error-free trials, the stimulus onset was determined based on a calculated mean of the error onsets from the error trials. Each epoch was baseline-corrected to the time span from 100 ms pre-stimulus to the stimulus onset. For feature extraction, the focus was on the specific temporal range from 0 to 800 ms after the stimulus onset, as it was considered to capture the essential aspects of the neural responses to the stimuli. The resultant data were structured into matrices; the error epochs were organized into a [64, 400, 48] matrix representing the number of channels, the number of time points within each epoch, and the total count of error epochs respectively, derived from eight training sets per participant, each containing six error epochs. Similarly, the error-free epochs were framed within a [64, 400, 192] matrix, drawn from eight sets each including 24 epochs.

**Figure 2:**
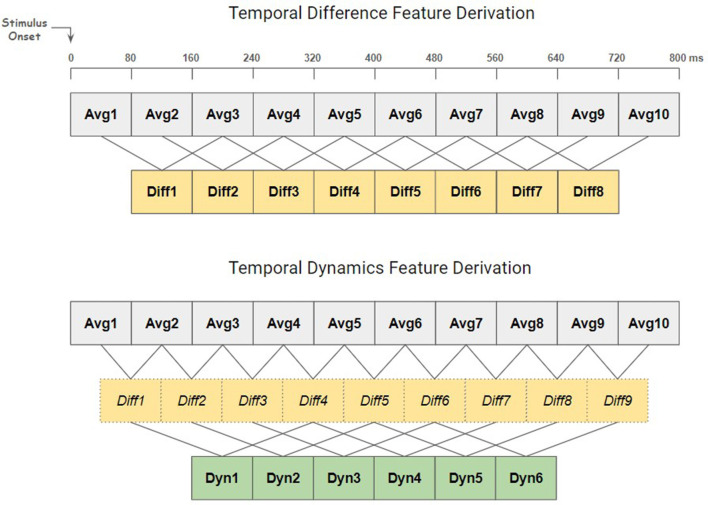
Derivation process of temporal derivative features. Top: the derivation of temporal difference (Diff) features, capturing variations over 160 ms intervals. Bottom: the derivation of temporal dynamics (Dyn) features, capturing variations over 400 ms intervals. Subtractive operations are indicated by lines. The temporal difference values in the bottom plot differ from those in the top plot and are only used to calculate the temporal dynamics values. Bold fonts indicate features that were used for classification.

According to the previous ERP analysis on one subject (Kueper et al., 2024), the averaging of epochs during error events revealed an ERP component peaking ~400 ms after the error. Building on this, the focus was directed toward the significant ERP component to efficiently extract temporal-spatial features from error epochs. Each epoch was partitioned into non-overlapping 80 ms windows and the mean value for each of these windows were calculated for all channels. This procedure generated the first type of features, termed “temporal average”. Furthermore, to enable a more comprehensive capture of the EEG signal's temporal characteristics, two special types of time-derivative features were introduced, termed “temporal difference” and “temporal dynamics” (see Figure 2). The temporal difference, representing the first derivative, was calculated for each window by subtracting the mean of the second preceding window (80 ms/one window gap between the two windows), enabling the detection of variations in EEG activity between non-adjacent time windows. This calculation effectively reveals the directional changes in the signal's amplitude and captures the transition between the valley and peak in the Pe component of the expected ERP. Temporal dynamics, on the other hand, entails calculating the second-order derivatives to reflect the rate of change in EEG activity between windows. To extract this feature, the first step was to derive temporal differences between directly adjacent original windows, unlike the process used to extract temporal differences above. Then, for each resulting temporal difference value, the third preceding temporal difference value was subtracted (160 ms/two windows gap between the two difference values) to obtain the temporal dynamics. This process was designed to map subtle fluctuations or trends that might not be visible through simply looking at the first-order derivatives. The dimensions of the extracted temporal-spatial feature matrices—representing channels and temporal features—are as follows: [64, 10] for temporal average, [64, 8] for temporal difference, and [64, 6] for temporal dynamics. All hyperparameters used in the feature extraction process, such as window length and step size, were determined based on the ERP analysis (Kueper et al., 2024), as well as on the cross-validation results of this competition. In particular the rise time and fall time of the ERP component were taken into account to determine the window size and step sizes for the differences and dynamics. While adopting smaller window lengths or step sizes might facilitate finer feature extraction, producing a rich set of features for selection, it may suffer the risk of overfitting. Therefore, it's important to consider the balance between parameter complexity and the model's ability to generalize to unfamiliar data when applying this methodology to other applications.

To gauge the discriminatory potential of each temporal-spatial feature, we calculated Cohen's *d* effect size, which offers a measure of the standardized difference between two groups. It is defined by the formula (Cohen, 2013):


$$\begin{array}{l} Cohen^{\prime }s\;d=\;\frac{Group\;A\;Mean-Group\;B\;Mean}{Pooled\;Standard\;Deviation} \end{array}$$

According to Cohen (2013), Cohen's *d* effect size has thresholds of large (*d* = 0.8), medium (*d* = 0.5), and small (*d* = 0.2). For each temporal-spatial feature extracted across the three different feature types, the absolute value of Cohen's *d* was calculated to quantify the effect size between event types (error/error-free). Features with a high Cohen's *d* value were recognized as having high discriminatory potential. Averaged Cohen's *d* topographies across all participants, based on 48 error and 192 non-error epochs per participant, were visualized to highlight the effect sizes across all feature types and their role in distinguishing between event types (see Figure 4).

**Figure 3:**
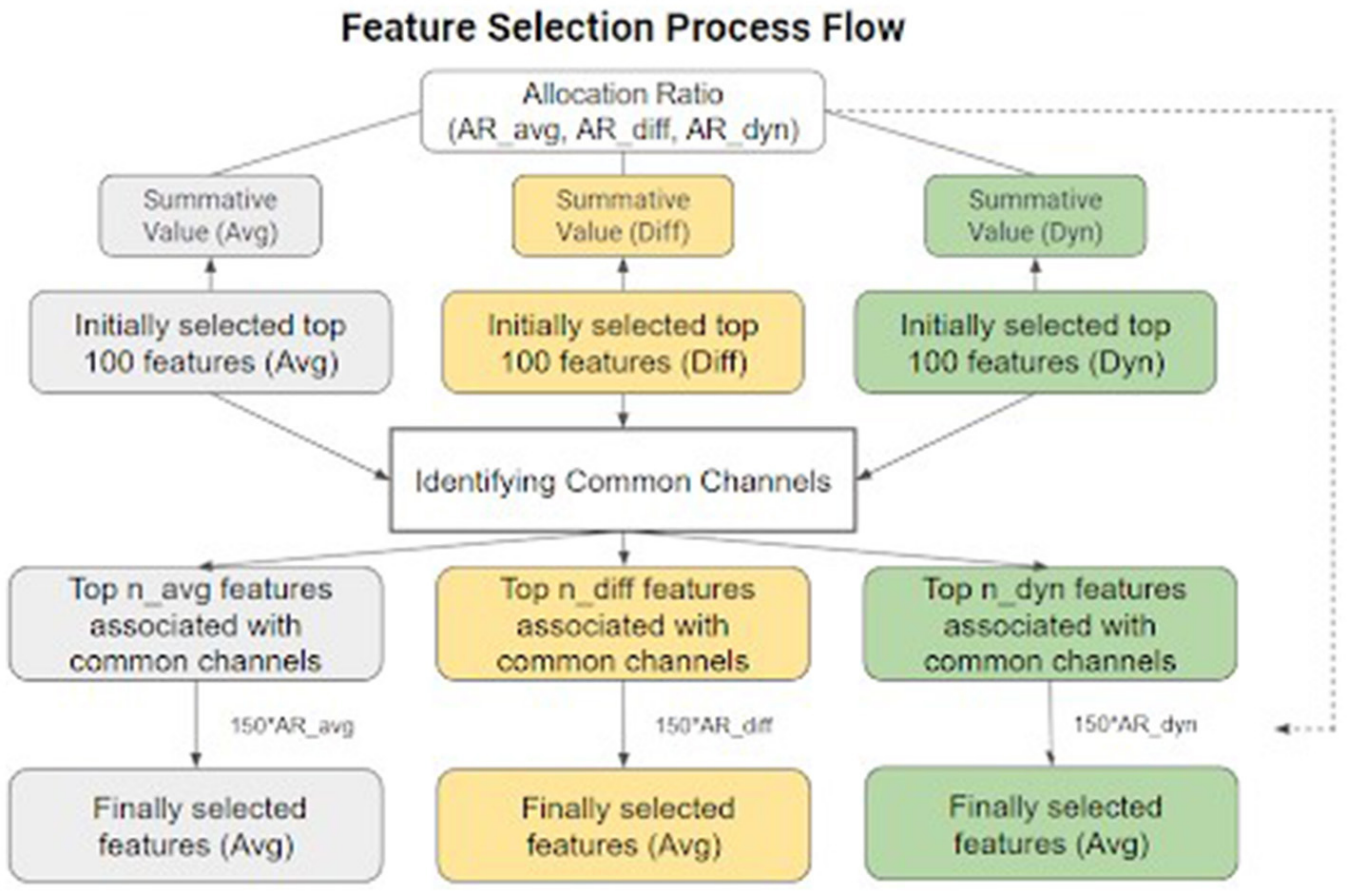
Feature selection process flow. Cohen's *d* was computed for each feature and then used to select common channels as spatial filters and for the final selection of 150 features used in the model.

As shown in Figure 3, the feature selection began with the identification of the top 100 features characterized by the highest absolute Cohen's *d* values for each kind of temporal feature individually. Subsequently, the 100 chosen values were aggregated separately for each feature type to obtain a summative value. The ratios of these accumulated values across the three feature types were calculated to represent their respective overall discriminatory abilities, a measure later referred to as allocation evidence in further processes. Thereafter, the channels that appeared in the top 100 features of all three feature types were identified as common channels. Only the features associated with these common channels were considered to assemble the final feature matrix, based on the idea that a valid ERP component identified in a single channel would consistently manifest high Cohen's *d* values across all three feature types. The feature selection process was finalized by choosing a total of 150 temporal-spatial features for further classification. They were distributed across the three feature types in only the pre-selected channels, based on the previously calculated allocation ratio, aiming to pinpoint the most discriminative temporal-spatial features for the upcoming phases.

To address the class imbalance issue, the Synthetic Minority Over-sampling Technique (SMOTE) was applied to enhance the representation of the minority class (error) by generating synthetic samples through interpolation with neighboring instances, thereby equalizing the number of samples in both classes within the feature matrix (Chawla et al., 2002). The resulting balanced feature set contained 384 epochs (192 error and 192 non-error epochs), each characterized by a 150-element feature vector. This dataset served as the input for training the Support Vector Machine (SVM) classifier (Cortes and Vapnik, 1995). The SVM model was configured with a linear kernel and a regularization parameter set to 1.0. To validate the model's performance, a 10-fold cross-validation was carried out individually for each participant. As the proposed feature selection method used the labels of the train sets, the train and test sets were split before the feature selection process to avoid overfitting. Each cross-validation iteration contained 24 epochs in the test set and 216 epochs in the train set per participant; after SMOTE interpolation, each balanced train set contained 346 epochs.

##### 2.5.1.2 Online simulation

During the online prediction simulation phase, a buffer-like moving window was used to simulate real-time data acquisition. Spanning 900 ms, this window covered the same time range used during the offline training phase for feature extraction (including 100 ms baseline range). With a step size of 20 ms, the window continuously “fetched” EEG data, ensuring a seamless and overlapping coverage of the incoming EEG data. The preprocessing approach and feature set chosen for classification in this phase were consistent with those applied in the offline training phase. Additionally, we implemented a lightweight noise-filtering method to evaluate the noise level in each window, termed the “Max-Min Amplitude Noise Filtering (MANF) Technique”. Using the error epochs in the training sets, a threshold was calculated by examining max-min amplitude differences. To this end, first, the max-min amplitude difference within each error epoch was calculated for each channel. Then, the mean of the top 10% max-min amplitude differences (six channels/values, consistently across all participants) was computed for each error epoch, and last, the mean of those means across all error epochs, plus one standard deviation, was set as the final noise threshold. Epochs that contained mean max-min amplitude differences in their top 10% channels above this threshold were deemed “noisy” and excluded from subsequent online simulation error prediction.

Finally, the trained SVM classifier continuously provided prediction scores for each data window, which represented the distance of individual samples to the decision hyperplane, thus indicating the confidence of it being classified as the error sample. According to the guidelines outlined in Section 2.3.1.2 for the offline stage, only 6 indices representing the predicted error onsets for each testing set needed to be submitted. The prediction scores were ranked, with the top 6 identified as the predicted errors. Based on the experiment rules set by Kueper et al. (2024), errors were introduced starting from the third trial and once an error appeared in a trial, it wouldn't reoccur in the subsequent two trials. This was used by choosing only those indices outside the estimated 7,000 ms range that covered two trials after an error was found. Prediction simulation was generated only within the estimated range from the third to the final trial. Additionally, to leave a margin and prevent predictions from occurring before the true onset of errors, the reported predictions were set to be 100 ms later than the initial predictions.

#### 2.5.2 Online stage

Before the online stage, the classifier was trained on the offline stage data again in Python 3.8.8, using all 10 available data sets and employing the same method as implemented in MATLAB. The trained model was then saved for use in the online stage. During the online stage of the competition, we participated remotely from Berlin, Germany, while EEG data for one participant was recorded and streamed via LSL from Bremen, Germany. Data was fetched every 40 ms using a 900 ms ring buffer. The data processing mirrored that of the training phase, and the SVM model and temporal-spatial feature selection were pre-loaded for the specified participant. The only change in signal processing compared to the offline stage was the use of a single 4th order Butterworth band-pass filter with cutoff values of 0.1 and 15 Hz (−3 db) for the band-pass filtering using the scipy.signal.butter function. A predetermined prediction threshold of 1.5 was used to identify errors, chosen for its conservative nature to minimize false alarms at the expense of increased misses. Upon detecting an error, its timestamp was promptly transmitted to the host via IP requests, along with the local LSL timestamp for calculating the computation duration.

#### 2.5.3 Extended analysis and evaluation of the methodology

##### 2.5.3.1 Comparative analysis and feature impact evaluation

To assess the effectiveness of the proposed time-derivative features and the associated feature selection technique, a comparative analysis of the offline training accuracy when using different feature types was performed using a reference method. This method involved the same pre-processing and epoching of the EEG data as the proposed approach. However, it used only traditional average features extracted using 50 ms non-overlapping moving windows (as used e.g., in Protzak et al., 2013, and Zander et al., 2016) within the (0–800) ms post-stimulus period across all 64 channels, yielding 1,024 features per epoch for classification. In line with our approach, an SVM was then trained using the SMOTE technique for class balancing and a 10-fold cross-validation was performed for each participant, providing a performance baseline for comparative analysis. In addition, we investigated the impact of window size on classification performance by examining the BA of both the proposed and reference methods under window lengths of 50 and 80 ms.

To investigate the impact of each feature type on classification accuracy, classifier performance evaluations were performed under five different conditions: using only temporal average, temporal difference, temporal dynamics, a combination of temporal average and temporal difference, and a combination of temporal average, temporal difference, and temporal dynamics. The feature derivation process followed the methodology outlined in Section 2.5.1.1, resulting in 640 temporal average features, 512 temporal difference features, and 384 temporal dynamics features. Feature selection was guided by Cohen's *d* values, although the selection process for the five conditions slightly differed from that shown in Figure 3. For each feature type, features were ranked based on their absolute Cohen's *d* values, and the top-ranked features were selected for input to the classifier. For combined feature types, the Cohen's *d* values were ranked collectively and the top-ranked features among them were selected. Finally, we computed the BA achieved with different numbers of selected features for each condition 20 times to obtain an average accuracy.

##### 2.5.3.2 *Evaluation of the MANF technique*

During the online simulation phase and the actual online stage, the MANF technique was used to identify noisy epochs. To assess the effectiveness of the MANF technique, the online simulation evaluation was repeated (see Section 2.5.1.2), but this time without the use of the MANF technique. We then compared the results from both conditions using the same evaluation metrics (see Section 2.3.1.2).

## 3 Results

### 3.1 Offline stage

#### 3.1.1 Classifier training

**Figure 4:**
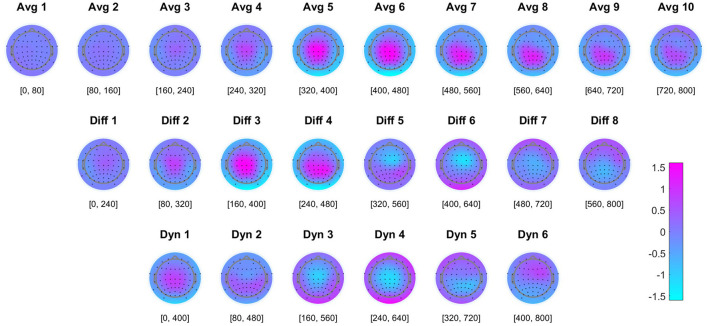
Feature effect size topographies. Averaged Cohen's *d* topographies for the different features in the offline training (*N* = 8 participants, with 48 errors and 192 non-error epochs per participant). Higher absolute values indicate a higher discriminability between the two classes (error/no error), with positive values corresponding to a positive deflection in the ERP amplitude. See Section 4.1 for details.

The averaged Cohen's *d* topographies across all participants are visualized (see Figure 4). Higher absolute values indicate greater discriminability between error and non-error event types, with positive values corresponding to a positive deflection in the ERP amplitude. The classifier's performance in the offline stage was evaluated in a 10-fold cross-validation. The average TPR, TNR, and BA are displayed (see Table 1). The average BA across all participants is 89.9%, significantly above the chance level of 50% accuracy (significance with α = 0.001 would have been reached with 73.68% correct classification, see Mueller-Putz et al., 2008).

**Table 1:** Offline training accuracies.

**Participant**	**TPR (Mean ±SD)**	**TNR (Mean ±SD)**	**BA (Mean ±SD)**
AA56D	0.7150 ± 0.2334	0.9379 ± 0.0404	0.8264 ± 0.1173
AC17D	0.8950 ± 0.1117	0.9737 ± 0.0277	0.9343 ± 0.0486
AJ05D	0.8750 ± 0.1439	0.9529 ± 0.0298	0.9139 ± 0.0831
AQ59D	0.9350 ± 0.1055	0.9789 ± 0.0368	0.9570 ± 0.0642
AW59D	0.8800 ± 0.1687	0.9847 ± 0.0341	0.9324 ± 0.0799
AY63D	0.7000 ± 0.2415	0.9474 ± 0.0555	0.8237 ± 0.1115
BS34D	0.8600 ± 0.2378	0.9950 ± 0.0158	0.9275 ± 0.1261
BY74D	0.7900 ± 0.2234	0.9634 ± 0.0498	0.8767 ± 0.1111
**Average**	**0.8313** **±0.0865**	**0.9667** **±0.0197**	**0.8990** **±0.0510**

Cross-Validation classification accuracies in the offline training (*N* = 8 participants, with 24 epochs in the test set and 346 epochs in the train set after SMOTE interpolation per participant in each cross-validation iteration). TPR, true positive rate; TNR, true negative rate; BA, balanced accuracy; SD, standard deviation.

#### 3.1.2 Online simulation

**Figure 5:**
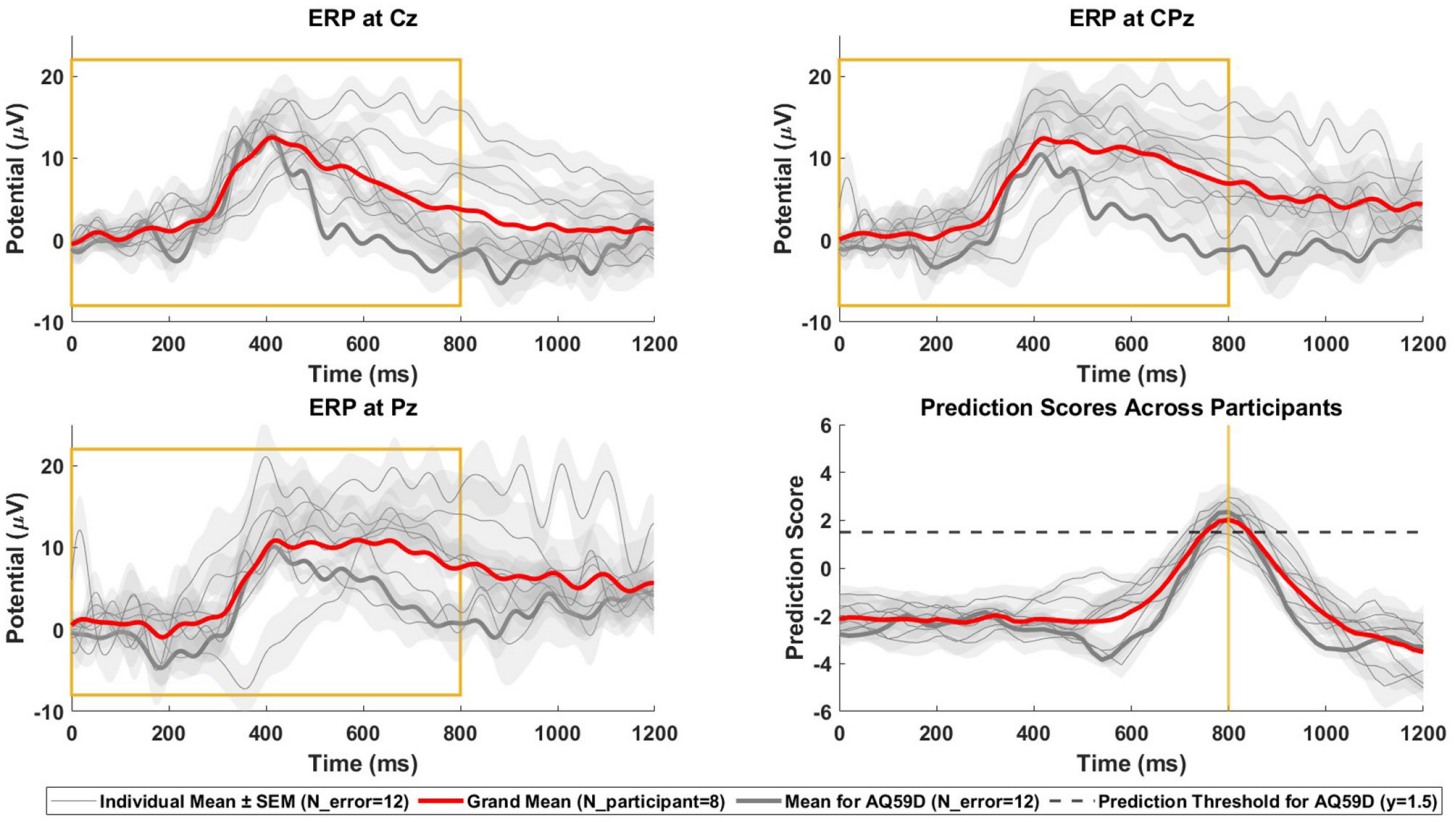
Online simulation ERPs and prediction scores. ERPs and prediction scores after error onsets in the online simulation analysis (*N* = 8 participants, two datasets with each *N*_error = 6 per participant). The figure presents the grand mean with red line, individual means with gray lines and the standard error of the individual means as shaded areas in each subplot. The thicker gray line highlighted within these represents the participant of the online prediction stage. **(Top-left)** ERPs at Cz. **(Top-right)** ERPs at CPz. **(Bottom-left)** ERPs at Pz. **(Bottom-right)** the prediction scores of the SVM classifier. The decision threshold for online error prediction is indicated by the dashed line. The yellow box highlights the window used for classification, aligned with the yellow line in the bottom-right plot, showing the peak prediction score for the data window (0–800) ms post-error, in correspondence with the window it was trained on.

**Figure 6:**
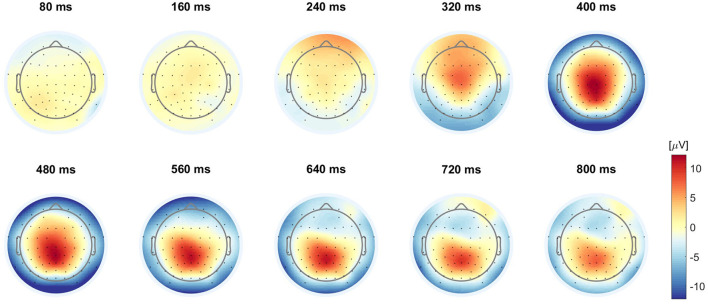
Grand averaged online simulation ErrP topographies. Grand averaged ErrP topographies in the online simulation of eight participants with 2*6 errors each (*N* = 96).

The final results of the online simulation, evaluated using the total set score and the number of TPs as described in Section 2.3.1.2, are presented in Table 2. This approach resulted in a total of 58 TPs out of a possible 96, translating to a 60.4% hit rate. The average temporal difference, calculated only from the correctly identified TPs, reached 104.0 ms, aligned with the 100 ms margin established to prevent predictions from occurring before the true onset of errors. Figure 5 illustrates the relationship between the EEG data used for prediction and the corresponding prediction scores. Specifically, it shows the ERPs at the Cz, CPz, and Pz electrode sites following error onset, together with the prediction scores derived from this data. Additionally, we display the grand average topographies following error onsets across eight participants in Figure 6.

**Table 2:** Results of online simulation.

**Participant**	**Set**	**Total set score (in ms)**	**No. of TPs**
AA56D	Set 5	2,660	4
	Set 6	2,570	4
AC17D	Set 5	2,248	4
	Set 6	2,438	4
AJ05D	Set 5	3,230	3
	Set 6	3,216	3
AQ59D	Set 6	1,426	5
	Set 7	2,380	4
AW59D	Set 5	1,572	5
	Set 6	1,610	5
AY63D	Set 5	2,448	4
	Set 6	2,392	4
BS34D	Set 5	4,210	2
	Set 6	5,018	1
BY74D	Set 5	3,368	3
	Set 6	3,244	3
**Sum**		**44,030**	**58**

Total Set Score refers to the cumulative temporal deviations across all 6 errors within the current set. Misses were counted with a deviation of 1,000 ms. True Positives (TPs) are defined as detections occurring within a 1,000 ms span following the error onset, with the maximum number of TPs per set being 6 (see Section 2.3.1.2 for further details).

### 3.2 Online stage

During the online stage, two scenarios were involved: one where participants were required to respond directly upon sensing an error, and another where no response was required, a condition not present during the classifier training phase. The results, assessed based on the evaluation metrics detailed in Section 2.3.2.2, are presented in Table 3. Table 3 shows that in both scenarios, a relatively high BA is observed, with 68.3 and 88.3% in the scenario with direct response, and 70 and 90% in the scenario without direct response. Notably, the TNR is substantially higher than the TPR. However, there is a notable decrease in the performance of the pre-trained classifier used for online classification compared to the offline training phase, where it achieved a TPR of 93.5%, a TNR of 97.9%, and a BA of 95.7%. Additionally, the performance exhibits variation across the session. Furthermore, the obtained *t*_score_ values are above 0.77 in all sets, indicating a swift error prediction, as the *t*_score_ value ranges between 0 and 1.

**Table 3:** Results of the online stage.

**Scenario**	**Set**	**TPs**	**FNs**	**TPR**	**FPs**	**TNs**	**TNR**	**BA**	**t_score_**	**FPS**
Direct response	Set 1	2	3	0.4	1	29	0.967	0.683	0.905	0.7496
	Set 2	4	1	0.8	1	29	0.967	0.883	0.8218	0.8646
No response	Set 1	2	3	0.4	0	30	1.0	0.7	0.845	0.7435
	Set 2	4	1	0.8	0	30	1.0	0.9	0.777	0.8631

TPs, true positives; FNs, false negatives; TPR, true positives rate; FPs, false positives; TNs, true negatives; TNR, true negatives rate; BA, balanced accuracy; *t*_score_ = consolidated time score according to Section 2.3.2.2; FPS, final performance score according to Section 2.3.2.2.

### 3.3 Extended analysis and evaluation of methodology

#### 3.3.1 Comparative analysis and feature impact evaluation

**Figure 7:**
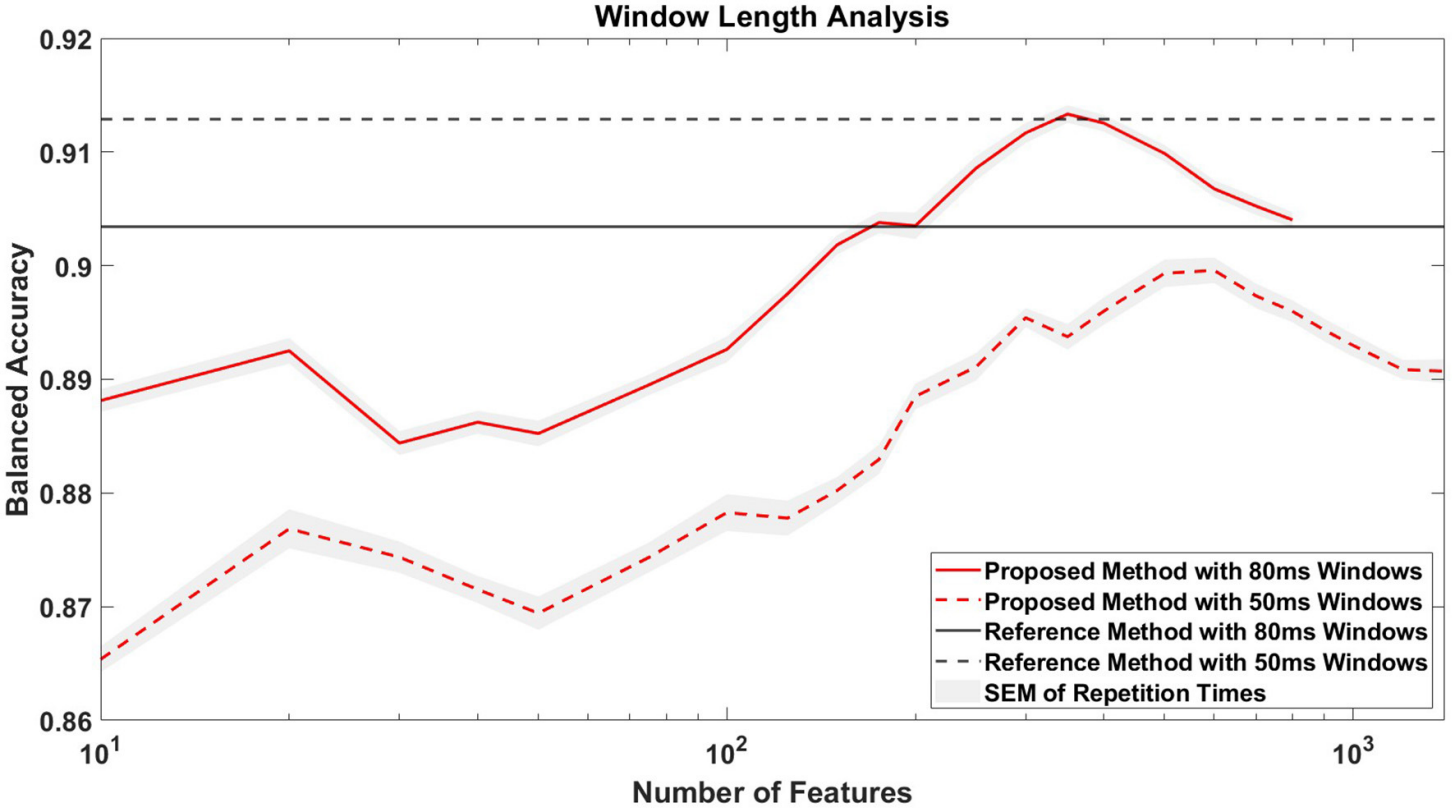
Window length analysis. The figure compares the classification accuracies of the proposed and reference methods for window lengths of 50 and 80 ms. The *x*-axis, logarithmized for clarity, represents the number of features selected in the proposed method. For context, the reference method uses 1,024 temporal average features when using 50 ms windows and 640 features when using 80 ms windows.

As described in Section 2.5.3.1, we used a reference feature extraction method and trained the SVM in the same way as the competition setup, and then evaluated it through 10-fold cross-validation for comparative analysis. The reference method using 1,024 average features achieved an average BA of 91.3 ± 4.5% (mean ± SD) across all participants, with an average TPR of 84.9 ± 7.8% (mean ± SD) and an average TNR of 97.7 ± 1.4% (mean ± SD). In contrast, our proposed method using 150 combined temporal features achieved an average BA of 89.9 ± 5.1% (mean ± SD), an average TPR of 83.1 ± 8.7% (mean ± SD) and an average TNR of 96.7 ± 2.0% (mean ± SD), which did not exceed the metrics of the reference method (see Table 1). Notably, Figure 7 shows that extending the window length to 80 ms in the reference method, thus reducing the number of features to 640, did not improve its performance, achieving an average BA of 90.4 ± 4.9% (mean ± SD) across all participants. However, when applying 80 ms windows in our proposed method, we consistently observed an approximate 2% increase in BA compared to the 50 ms window. It is worth noting that with 350 selected features and 80 ms windows, the proposed method matched the BA of the reference method that used 50 ms windows and all 1,024 features.

**Figure 8:**
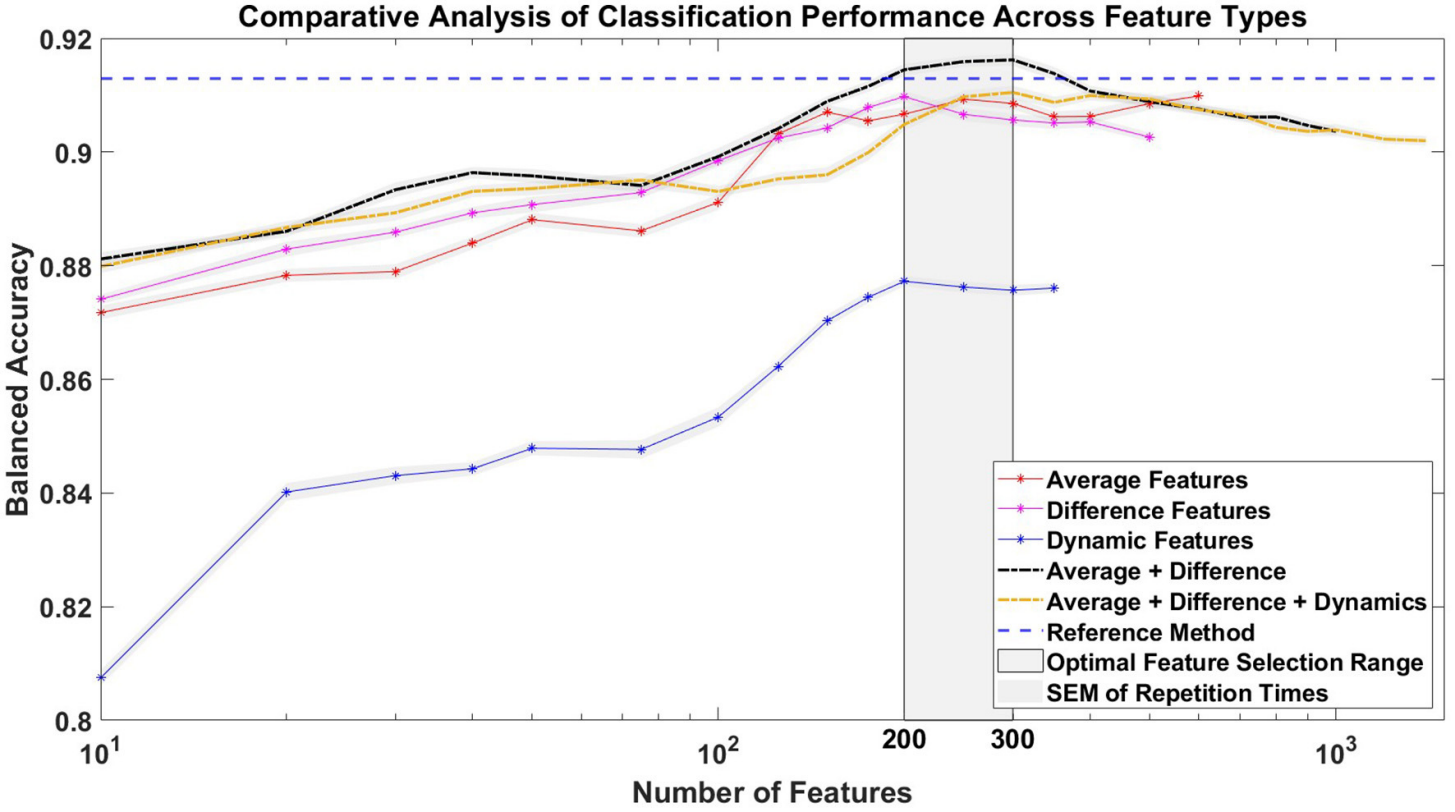
Impact of feature types on average balanced accuracy (BA). The figure shows the classification accuracy achieved with different numbers of selected features under five different conditions: using only temporal average features, temporal difference features, temporal dynamics features, a combination of temporal average and temporal difference features, and a combination of temporal average, temporal difference, and temporal dynamics features. Feature selection was guided by the ranked Cohen's *d* values. For comparison, the average BA of the reference method, which uses 1,024 temporal average features, is shown as a horizontal line. The gray zone indicates the ideal range for selecting the number of features, specifically between 200 and 300, which is associated with achieving the highest BA. The *x*-axis, representing the number of features, is logarithmized.

In addition, Figure 8 illustrates the impact of each feature type on the classification accuracy with different numbers of selected features. Temporal difference features showed comparable classification performance, although not better than temporal average features. The combined use of temporal average features and temporal difference features slightly improved the BA compared to the reference method, but only when the number of features used was between 200 and 350. The use of temporal dynamics features alone resulted in a BA ~4% lower than the use of temporal average or temporal difference features alone, and the integration of temporal dynamics features with the other two feature types actually decreased the BA. Notably, the optimal BA for any combination of feature types was not achieved by using all features, but rather with a selection of ~200–300 features, as determined by ranked Cohen's *d* values.

#### 3.3.2 Evaluation of the MANF technique

The results of the comparative analysis between the condition using the MANF technique and the condition without the MANF technique are shown in Table 4. The cumulative total set score serves as a comprehensive metric to evaluate the performance in error detection and FA rejection, allowing a qualitative comparison between the two conditions. In general, a higher cumulative total set score indicates fewer correct detections and more FAs. The MANF technique produced a lower cumulative total set score, which translated into a higher number of TPs with 58 out of a possible 96, giving a hit rate of 60.4% compared to the condition without the MANF technique, which had a higher cumulative total set score of 54,196 ms and a lower hit rate of 49.0%.

**Table 4:** Comparative analysis between the condition using the MANF technique and the condition without the MANF technique.

**Noise filtering condition**	**Cumulative total set score (in ms)**	**Total no. of TPs**
Without the MANF technique	54,196	47
Using the MANF technique	44,030	58

Cumulative total set score refers to the cumulative temporal deviations across all 12 errors within the two test sets across all participants. Misses were counted with a deviation of 1,000 ms. True Positives (TPs) are defined as detections occurring within a 1,000 ms span following the error onset, with the maximum number of TPs per set being 6 (see Section 2.3.1.2 for further details).

## 4 Discussion

In this study, an approach for online BCI-based machine error detection in the continuous EEG was proposed and applied in a human-machine interaction experiment as a winning strategy in a challenge at the IJCAI 2023 conference. This approach effectively combined three types of temporal features with an effect size-based feature selection strategy for model training, and was further enhanced by a lightweight noise filtering technique, specifically designed for non-calibrated online sessions. Extended research was conducted to validate the feasibility of the proposed methods. Offline training resulted in high cross-validation accuracies of 89.9% and a simulated online test resulted in a good hit rate on continuous data. Furthermore, despite a performance drop, the classifier maintained a high BA in the online continuous classification 3 months after training without recalibration, achieving high BAs across all sets while maintaining a fast error prediction capability.

### 4.1 Classification performance in the competition

In the offline stage of the competition, the average BA during cross-validation on the training sets was 89.9 ± 5.1% (mean ± SD) across all participants, indicating a good fit to the training data. The average TPR was 83.1 ± 8.7% (mean ± SD), which was lower than the average TNR of 96.7 ± 2.0% (mean ± SD). This discrepancy suggests a relatively weaker performance in identifying errors compared to rejecting FAs. There are two reasons for this: First, due to the high probability of FAs in continuous online classification, a conservative strategy was taken to minimize FAs, potentially at the expense of missing some errors. This involved setting a conservative error detection threshold and implementing a noise filtering technique. Secondly, the limited number of error epochs available for training, coupled with the limitations of SMOTE to address class imbalance, further contributed to the challenge of accurate error detection. Furthermore, our proposed method in the competition did not outperform the reference method in terms of metrics (see Section 3.3.1). However, when using 80 ms windows and 350 selected features, the proposed method achieved a slightly higher BA than the reference method, which used a 50 ms window and all 1,024 average features. We believe the reason for this result lies in optimizing window size and feature selection. Longer window sizes and a condensed feature set can increase the relevance of the data and reduce overfitting, thereby enhancing the overall efficiency of the classifier. We then simulated real-time data acquisition on the test sets using a buffer-like moving window that progressed through the EEG data. The trained SVM classifier produced prediction scores for each of these moving windows and the top six prediction scores were chosen for each test set containing six errors. This resulted in a total set score of 44,030 ms, corresponding to a sum of 58 TPs and a hit rate of 60.4%. Although the model's efficiency in detecting errors decreases compared to its performance during the training phase, this decrease should be contextualized by the application on continuous data and the competition rule that limited submissions to the top six errors per test set. Such a restriction inevitably led to missed errors that did not fall within these top six ranks. The grand average curve of prediction scores across all participants peaks ~800 ms after the onset of the error, coinciding with the moment when the buffer fetches EEG data from 0 to 800 ms post-error for prediction (see Figure 5). In terms of the model's ability to detect errors in continuous data, even the participant with the lowest prediction score peak achieved a mean score of 0.91 at the moment of error occurrence. In contrast, the grand mean of prediction scores outside the time range from 600 to 1,000 ms post-error consistently remains below zero, underscoring the classifier's ability to accurately classify them as error-free. In real-time applications, it is advisable to establish a unique decision threshold for SVM prediction scores for each individual, with prediction scores above the threshold being identified as errors. For the online stage, we chose 1.5 as a conservative threshold. In sum, our model was successful in detecting errors close to their actual time of occurrence in the simulated online test.

To confirm that our model relied on neurophysiological patterns rather than artifacts for simulated online classification, we visualized the grand averaged online simulation ErrP topographies, and compared it to the average Cohen's *d* topographies from the training sets across all participants (see Figure 4). Haufe et al. (2014) pointed out that patterns of forward models in EEG or other neuroimaging analyses have meaningful neurophysiological interpretations. Given that the presented Cohen's *d* topographies provide a statistical representation of the differences between two classes as derived from the output of the forward model, we can claim that these topographies reflect the sources of neural activity that are relevant to our study and can be used in the context of other research. As shown in Figure 4, in the first row, higher absolute Cohen's *d* values, which indicate a higher discriminability between the two classes (error/no error) and are used for feature selection, closely match with the centro-parietal positive deflection of the ERP shown in Figure 6. This congruence between averaged Cohen's *d* topographies and averaged ERP topographies supports our model's use of neurophysiological patterns for classification rather than artifacts.

In the online stage of the competition that happened 3 months later, the pre-trained model of one participant without any additional calibration or tuning was applied to the same participant doing the same experiment again, but with a new scenario of no physical response to errors. Here, similar accuracies were obtained in both scenarios, indicating that the performance of our model was primarily driven by the detection of ErrPs, rather than by motor preparation. Consistent with the offline training results, the TPRs were much lower than the TNRs in all sets, again due to our conservative strategy and the issue of class imbalance. While the TNRs remained consistent from the offline training session to the online classification session, we observed significant variability in TPRs during the online session for both scenarios, specifically 40% in set1 and 80% in set2. This intra-session variability was likely due to differences in ERP morphology; the ERP morphology in the offline session closely resembled that in set2 of the online session, but diverged from that in set1. This observation indicates that the proposed feature selection method is highly sensitive to variations in ERP morphology. Additionally, the reduction in TPRs between the offline session and the online session 3 months later may be attributed to EEG cross-day non-stationarity, a phenomenon also documented in other studies (Shenoy et al., 2006; Christensen et al., 2012; Krumpe et al., 2017). Furthermore, the obtained *t*_score_ values are above 0.77 in all sets. For this metric, which is in the (0,1) range, a higher score corresponds to a shorter duration between the true error stimuli and the real-time identification of the error. This score embodies both the efficacy of the model and the computational duration involved as we indicated both the estimated true error onset as well as the time that was required for our model to arrive at this decision. Our model used a buffer window of 800 ms and the prediction score rose above 1.5 around the 750 ms mark post-error (see Figure 5). We subtracted 800 ms from the time of this prediction score above 1.5 to arrive at an estimate of the true error onset. Prematurely detected errors were discarded according to the rules, so we can assume that the model caught most errors without any relevant delay. The computation time for the SVM model was in the range of 20 ms on our machine. The most relevant aspect contributing to a delay in detecting errors is thus the fact that our model requires a buffer window of 800 ms and can only detect an error around that time after it occurred. Depending on the use case, this might be an acceptable situation, but it could be explored whether a decrease in the buffer width results in a lower delay while maintaining a high prediction accuracy. In sum, our model was effective in detecting 60% of errors in an online application without almost no FAs even months after training, and it did so in a timely manner, most likely <1 s after the error occurred.

### 4.2 Feasibility of using time-derivative features and effect size-based feature selection technique in EEG-based classification

In this study, we hypothesized that a combination of three different feature types, including two time-derivative features and the traditional averaged amplitude features would more effectively capture the pattern of ERPs and distinguish them from artifacts with different patterns. However, our results showed that although the proposed time-derivative features were effective in predicting errors, they did not outperform traditional average features. The temporal difference features showed a comparable capacity in feature representation, but did not exceed the effectiveness of the temporal average features. The combined use of temporal average features and temporal difference features resulted in only a marginal improvement in the BA when using a certain number of features. Using temporal dynamics features alone resulted in lower BA compared to the other two types, suggesting that incorporating this feature type into the classification may not be beneficial as it led to decreased BA. The limited improvement of EEG-based classification performance by time-derivative features can be attributed to the nature of information they encapsulate. Most of the information relevant for classification is already captured by the temporal average features, which explains why the addition of derivative features may not substantially improve performance. In particular, second-order derivatives may add even less value due to their higher redundancy compared to first-order derivatives, as well as their sensitivity to noise in the data. In addition, classifiers such as SVMs are adept at interpreting and extracting relevant information from average features, which reduces the potential added value of time-derivative features. Therefore, the effectiveness of incorporating different types of features into classification models is highly dependent on the uniqueness of the information that each feature contributes. If the features are highly correlated or convey analogous information, their combined use may not lead to a substantial improvement in performance. To this end, when integrating different feature types for classification or in cases of modality fusion, it is crucial to ensure that each feature type or modality provides complementary information to enrich the model with new insights and dimensions.

Furthermore, we used Cohen's *d* values for feature selection. As shown in Figure 8, the highest BA for any combination of feature types was not achieved by using all features. Instead, optimal results were achieved with a subset of ~200–300 features, as identified by the ranked Cohen's *d* values. Compared to the reference method using 1,024 temporal-spatial features, this feature selection method can efficiently identify the most discriminative temporal-spatial features, reducing the feature dimension to one-fifth of its original size in this study. Moreover, the classification accuracy remained impressively high, exceeding 87% for most conditions, even with an extremely reduced feature dimension of only 10 features. This observation highlighted the effectiveness of our feature selection method in substantially reducing the feature dimension without much loss of classification performance. Lotte et al. (2018) highlight that precise feature selection, by reducing the number of features, leads to a reduction in the parameters that need to be optimized by the classifier. This reduction not only increases computational efficiency, allowing faster predictions for new samples, but also simplifies the identification of features that are truly relevant to the mental states under consideration. In real-time scenarios, where fast computation is essential for the viability of passive BCI-based applications, the development of more efficient feature extraction strategies is crucial. The feature selection method based on Cohen's *d* demonstrated its effectiveness in the continuous online classification in the competition. In addition, as shown in Figure 6, the visualization of the discriminability between two classes, represented by Cohen's *d* in the topographies, effectively identifies the temporal-spatial features that make a great contribution to the ErrP-based classification. This ensures that the classification is truly neurophysiologically based and is not biased by artifacts. In summary, this feature selection method is able to select the most discriminative temporal-spatial features, effectively reducing the feature dimension, which could be beneficial in time-critical circumstances or when computing on low-end hardware. Furthermore, it also provides a convenient way to investigate the neurophysiological background of the ongoing phenomenon.

### 4.3 Offline to online session transfer

Regarding the transfer from offline to online sessions, we first addressed the issue of potentially high FAs in real-time applications by implementing a simple noise filtering technique. This method, based on amplitude variability, effectively minimized FAs caused by large artifacts. Due to its minimal computational complexity, this technique is expected to perform efficiently in real-time scenarios, although a detailed computational time analysis has not been conducted within this study. However, it is particularly suited to controlled scenarios, such as situations where participants remain relatively stationary, as it does not correct for artifacts, but rather excludes entire epochs that are considered noisy.

In addition, our study shows how classification performance in online sessions can be affected by EEG non-stationarity, especially when no pre-calibration is performed before using an offline pre-trained classifier. Although our approach achieved a remarkable BA in online classification, we observed a decrease in TPR compared to the offline session and variability in TPR even within the same online session, likely due to cross-day and intra-session variability in EEG signals. These findings not only confirm known challenges, but also highlight the complexity of the transfer from offline to online sessions in BCI systems.

### 4.4 Limitations and future work

While our study shows considerable potential, it has certain limitations that require further investigation. First, the proposed method in the competition did not achieve optimal performance compared to the reference method, mainly due to time constraints during the competition and non-optimal choices of hyperparameters such as number of features. Therefore, a more in-depth study of time-derivative features and effect size-based feature selection technique was conducted after the competition to improve the proposed approach. Regarding the online application, while our approach proved to be feasible for continuous online classification during the competition, we had no opportunity to conduct a comparative analysis with other existing methods in an online context after the competition. As a result, the advantages of our approach for online scenarios in feature dimension reduction remain insufficiently explored. Second, additional research in different experimental contexts, real-world scenarios, and different datasets is needed to thoroughly assess the feasibility of the proposed approach. In particular, while the proposed time-derivative features did not outperform traditional amplitude average features in this study, results may vary across different datasets or applications. In addition, the feature selection method using Cohen's *d* showed promise in selectively identifying the most discriminative temporal-spatial features and substantially reducing feature dimensionality in this study, but future studies on standard datasets are needed to evaluate its effectiveness in improving classification performance. Furthermore, the MANF technique, which is effective in controlled scenarios where participants are mostly stationary, does not really remove the artifacts but excludes entire noisy epochs, potentially leading to missed detections in noisy environments. This highlights the clear need for future research to develop lightweight artifact removal techniques suitable for continuous online classification scenarios where fast computation is critical. Finally, to better accommodate the transfer from offline to online sessions, the development of adaptive classifiers capable of tracking potentially changing feature distributions is essential to maintain effectiveness amidst the non-stationarity of EEG signals (Lotte et al., 2018). In all, our study serves as a feasibility research concerning these aspects, while paving the way for future investigations to address these limitations, with the aim of developing more robust and adaptable BCI systems for continuous online error detection.

## 5 Conclusion

This study presents a comprehensive approach for continuous online machine error detection during a human-robot interaction task, integrating two time-derivative features, an effect size-based feature selection technique for model training, and a lightweight noise filtering method suitable for online sessions. The approach yielded a 89.9% accuracy during calibration, a 60.4% hit rate in online simulation, and an averaged accuracy of 79.2% in online tests conducted 3 months later without recalibration, while maintaining a low FA rate of 1.7%. In addition, a detailed analysis was performed to further validate the proposed method. Although time-derivative features were effective in predicting errors, they did not outperform traditional average features. In particular, the effectiveness of first-order time-derivative features was found to be equivalent to that of time-averaged features, while second-order features did not show the same level of effectiveness. The combined use of these features resulted in only a slight improvement in BA within a certain selected feature number range. The feature selection method based on Cohen's *d* not only efficiently identified the most discriminative temporal-spatial features, but also facilitated the exploration of the neurophysiological basis of the observed phenomena. The noise filtering technique designed for online sessions proved highly effective in minimizing FAs. The study also sheds light on the challenges posed by EEG cross-day and intra-session variability on classification performance in online sessions, highlighting the complexities involved in the transfer from offline to online sessions in BCI systems. Going forward, comprehensive research in different experimental settings, real-world scenarios and across different datasets is imperative to fully establish the practicality of the proposed approach. Furthermore, there is an urgent need for further studies aimed at developing more robust and adaptable BCI systems for continuous online error detection.

## Scope statement

Our manuscript contributes to the “Neurotechnology and Systems Neuroergonomics” section of Frontiers in Neuroergonomics, aligning with core themes such as brain-computer interfaces and neuroadaptive systems. It presents an advanced EEG-based error detection approach that is crucial for human-robot interaction in neuroadaptive technology. Our research makes two significant contributions to the field. Firstly, it demonstrates the feasibility of integrating two temporal derivative features with an effect size-based feature selection strategy, particularly in online EEG-based BCIs. Secondly, our work introduces an innovative approach designed for continuous online error prediction, which includes a straightforward noise rejection technique to reduce false alarms. Given this, our approach addresses essential facets such as signal preprocessing, feature extraction and machine learning, and real-time computing techniques in neuroergonomics applications. This manuscript underlines our commitment to research in the development of neuroergonomic systems and interfaces, and thus fits perfectly with the scope of the journal.

## Data availability statement

Publicly available datasets were analyzed in this study. This data can be found here: https://zenodo.org/records/8345429.

## Ethics statement

The studies involving humans were approved by local Ethical Committee of the University of Duisburg-Essen, Germany. The studies were conducted in accordance with the local legislation and institutional requirements. The participants provided their written informed consent to participate in this study.

## Author contributions

YP: Conceptualization, Methodology, Software, Writing – original draft, Writing – review & editing. TZ: Supervision, Writing – review & editing. MK: Supervision, Writing – review & editing, Writing – original draft, Methodology.

## Footnotes

^1^ https://ijcai-23.dfki-bremen.de/competitions/inter-hri/

^2^ https://zenodo.org/records/8345429
